# A recycling culture of *Neochloris oleoabundans* in a bicarbonate-based integrated carbon capture and algae production system with harvesting by auto-flocculation

**DOI:** 10.1186/s13068-018-1197-6

**Published:** 2018-07-24

**Authors:** Chenba Zhu, Ruolan Zhang, Longyan Cheng, Zhanyou Chi

**Affiliations:** School of Life Science and Biotechnology, Dalian University of Technology, Dalian, China

**Keywords:** Bicarbonate, Flocculation, Recycling culture, *Neochloris oleoabundans*

## Abstract

**Background:**

A bicarbonate-based integrated carbon capture and algae production system (BICCAPS) uses carbonate to capture CO_2_ and produce bicarbonate for alkalihalophilic microalgal cultivation. In this process, carbonate is regenerated and re-used for CO_2_ capture. However, a practical example of a recycling culture to prove its feasibility is still absent.

**Results:**

To reach this goal, a recycling culture of *Neochloris oleoabundans* was created in this study. The effect of bicarbonate concentration on *N. oleoabundans* growth showed that the highest productivity was obtained at 0.3 mol L^−1^, but the highest apparent carbon utilization efficiency was obtained at 0.1 mol L^−1^. The harvest of algal biomass was tested with alkaline flocculation, which is induced by high pH due to bicarbonate consumption. The result showed that the maximum recovery rate of 97.7 ± 0.29% was reached with a supplement of 20 mM Ca^2+^. Compared with this, alkaline flocculation without Ca^2+^ also resulted in a high recovery rate of up to 9 7.4± 0.21% in culture with 0.7 mol L^−1^ bicarbonate. In recycling culture, the spent medium was bubbled with CO_2_ and re-used for algal culture. After eight times of recycling, biomass productivity in recycling culture with 0.1 and 0.3 mol L^−1^ bicarbonate was 0.24 and 0.39 g L^−1^ day^−1^, respectively, higher than the 0.20 and 0.30 g L^−1^ day^−1^ in the control. The apparent carbon utilization efficiencies achieved in these semi-continuous cultures with 0.1 mol L^−1^ bicarbonate were 242 ± 3.1 and 266 ± 11% for recycling and control culture, respectively, while those with 0.3 mol L^−1^ bicarbonate were 98 ± 0.78 and 87 ± 3.6%, respectively.

**Conclusions:**

This study proved the feasibility of BICCAPS recycling culture with the first practical example. More importantly, the produced algal biomass can be harvested without any flocculant supplement. Thus, this process can reduce both culturing and harvesting costs in algal biomass production.

## Background

Worldwide CO_2_ emissions have increased the annual average concentration in the atmosphere to 400 ppm [1], and this has resulted in a series of problems, including global warming and ocean acidification. This situation may be mitigated by replacing fossil fuels with renewable energy or recycling utilization of CO_2_ [2]. Microalgae are promising to contribute to both of these two approaches by converting CO_2_ into biofuel [3]. However, commercial production of microalgal biomass is still limited by high costs [4, 5]. A process named bicarbonate-based integrated carbon capture and algae production system (BICCAPS) was proposed to reduce algal biomass production cost [6]. In this system, bicarbonate generated from CO_2_ absorption by carbonate is used to culture alkalihalophilic microalgae to avoid the high cost of CO_2_ purification and transportation. At the same time, it uses carbonate regenerated from algal culture to absorb CO_2_ and to reduce the high cost of carbonate regeneration with traditional methods [6]. Thus, BICCAPS is promising to simultaneously reduce the cost of carbon capture and microalgal culture.

To test the feasibility of BICCAPS, alkalihalophilic cyanobacterium *Euhalothece* sp. was cultured with 1.0 M sodium bicarbonate. This culture resulted in an algal biomass productivity of up to 1.2 g L^−1^ day^−1^, indicating that high productivity is achievable at this extreme condition [7]. This high concentration of bicarbonate can supply sufficient carbon at the beginning of each culture, with no continuous CO_2_ bubbling or interval feeding necessary. This allows the use of a photobioreactor with a simple structure. A low-cost horizontal floating photobioreactor without gas bubbling and/or an agitation system that uses waves as the only energy for mixing was developed in our previous study [8]. This progress systematically reduced the cost of photobioreactor manufacturing, carbon supply, energy consumption, and culture condition control (pH, DO, temperature) [8] and showed great potential to reduce microalgal biomass production cost. However, recycling the culture is obligatory for a BICCAPS, since sodium bicarbonate has a price of approximately $200 ton^−1^, and it would be more expensive than using CO_2_ as feedstock if not recycled. Thus, the feasibility of using spent medium enriched with carbonate to absorb CO_2_ and conducting culture recycling must be proven by experiment.

In addition to cultivation, the harvesting of algal biomass is a significant cost and accounts for approximately 20–30% of total production cost [9]. Flocculation is promising to reduce this cost, but it is usually induced by a flocculant supplement [10]. This is not only expensive but also causes the potential problem of biomass contamination [11]. To address these problems, alkaline flocculation (auto-flocculation) was proposed as a simple method [12]. It is induced by a high pH and the precipitation of calcite (calcium carbonate) or brucite (magnesium hydroxide) [13]. However, flocculation induced by calcite is not stable. Studies have shown that although high flocculation efficiency was achieved with 12.5 mM calcium concentration [13], extensive CaCO_3_ precipitation yielded only partial flocculation in another study [14]. Different from this, magnesium hydroxide-induced flocculation worked well in a high pH range of 10–11 [15]. However, this requires a large amount of alkaline to increase the pH and it is very expensive [11].

**Fig. 1:**
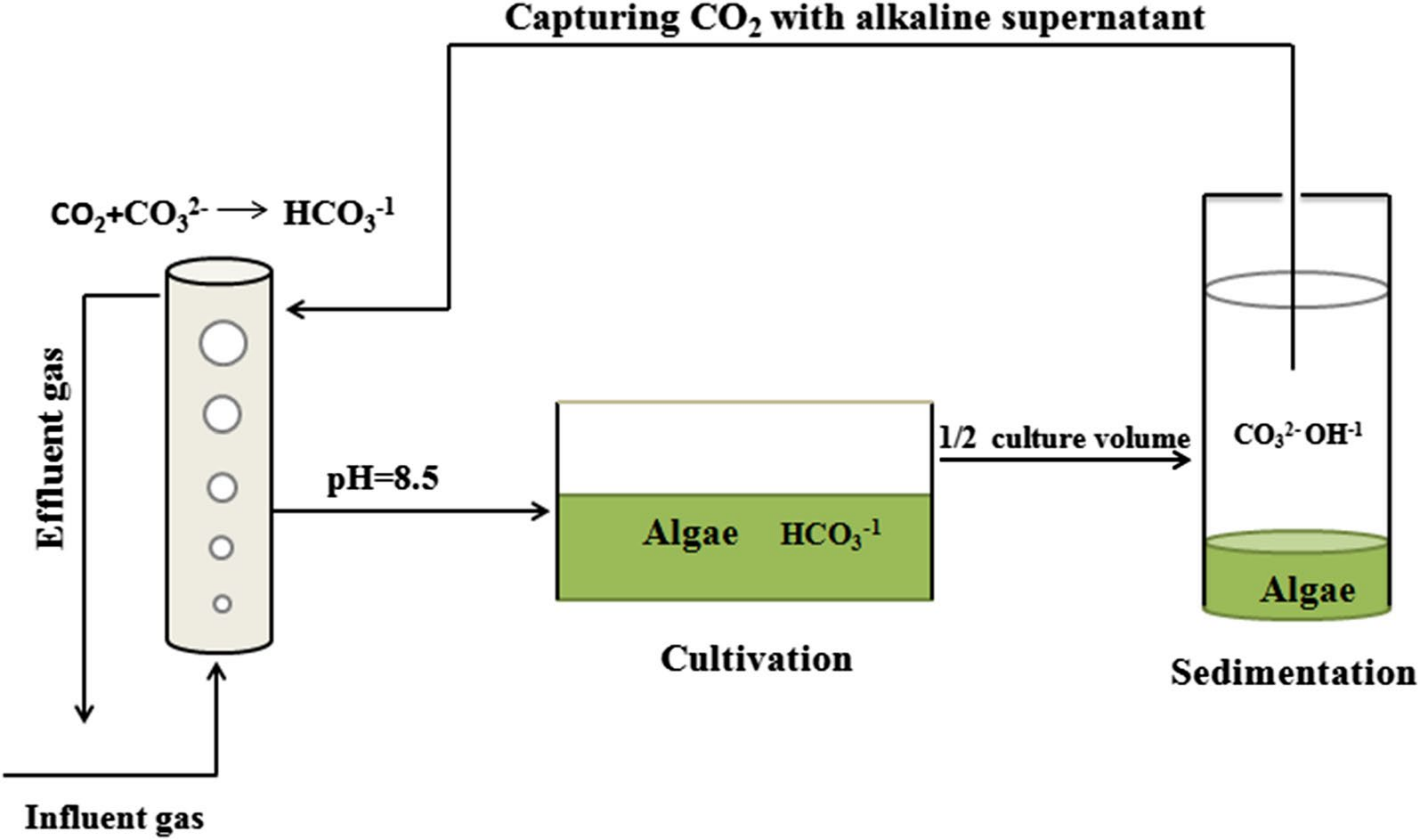
Closed-loop bicarbonate/carbonate recirculation for algal cultivation, harvesting, and carbon capture in BICCAPS

In a BICCAPS, consumption of HCO_3_^−^ leads to an increased pH and a higher ratio of CO_3_^2−^/HCO_3_^−^ [16]. This may be used to induce auto-flocculation by adding low concentrations of calcium, or ideally, without any flocculant supplement (Fig. 1). Thus, auto-flocculation was tested in the harvesting of algal biomass in this study. *Neochloris oleoabundans* was selected for this test, since it is tolerant to high concentrations of bicarbonate [17] and its biomass is enriched with lipid for biofuel production [18, 19]. However, only alkalihalophilic microalgae are suitable for BICCAPS. Thus, selection of alkalihalophilic strains is very important. It was reported that some microalgal strains in soda lake evolved to be tolerant to saturated bicarbonate [20]. Thus, an adaption process was conducted to improve algal strains’ tolerant capability to high concentration of bicarbonate at first, and this was achieved by gradually increasing bicarbonate concentration contained in the medium. Culture recycling of *N. oleoabundans* was conducted with a semi-continuous mode, in which spent medium was used to absorb CO_2_ and then re-used in cultivation. The results reported here proved the feasibility of BICCAPS culture recycling with a practical example.

## Methods

### Microalgal strain and culture medium

The microalgal strain *N. oleoabundans* UTEX1185 was purchased from the Culture Collection of Texas University (USA). It was cultivated in medium that contained NaHCO_3_, 25.2 g L^−1^; NaNO_3_, 0.5 g L^−1^; NaCl, 0.025 g L^−1^; MgSO_4_, 0.05 g L^−1^; KH_2_PO_4_, 0.322 g L^−1^; CaCl_2_, 0.02 g L^−1^; FeCl_3_·6H_2_O, 0.005 g L^−1^; and A5 trace elemental solution 1 mL L^−1^ [17]. However, at first, the algae could not grow well in culture medium containing 0.3 mol L^−1^ bicarbonate, although it was reported that this algae can tolerate 0.3 mol L^−1^ bicarbonate [17]. Thus, the algae were first cultured with 0.08 mol L^−1^ bicarbonate, and then, the concentration was increased by 0.02 mol L^−1^ every 4 days until it reached 0.3 mol L^−1^. This culture was conducted in a 1-L Erlenmeyer flask with a work volume of 0.4 L, and it was bubbled with air at a rate of 100 mL min^−1^. Every 4 days, 50% of the medium was replaced with fresh medium containing 0.08 mol L^−1^ bicarbonate. This process lasted for 6 months, and the obtained algal strain adapted to a NaHCO_3_ concentration of 25.2 g L^−1^, or 0.3 M.

### Algal cultivation and biomass concentration measurement

**Fig. 2:**
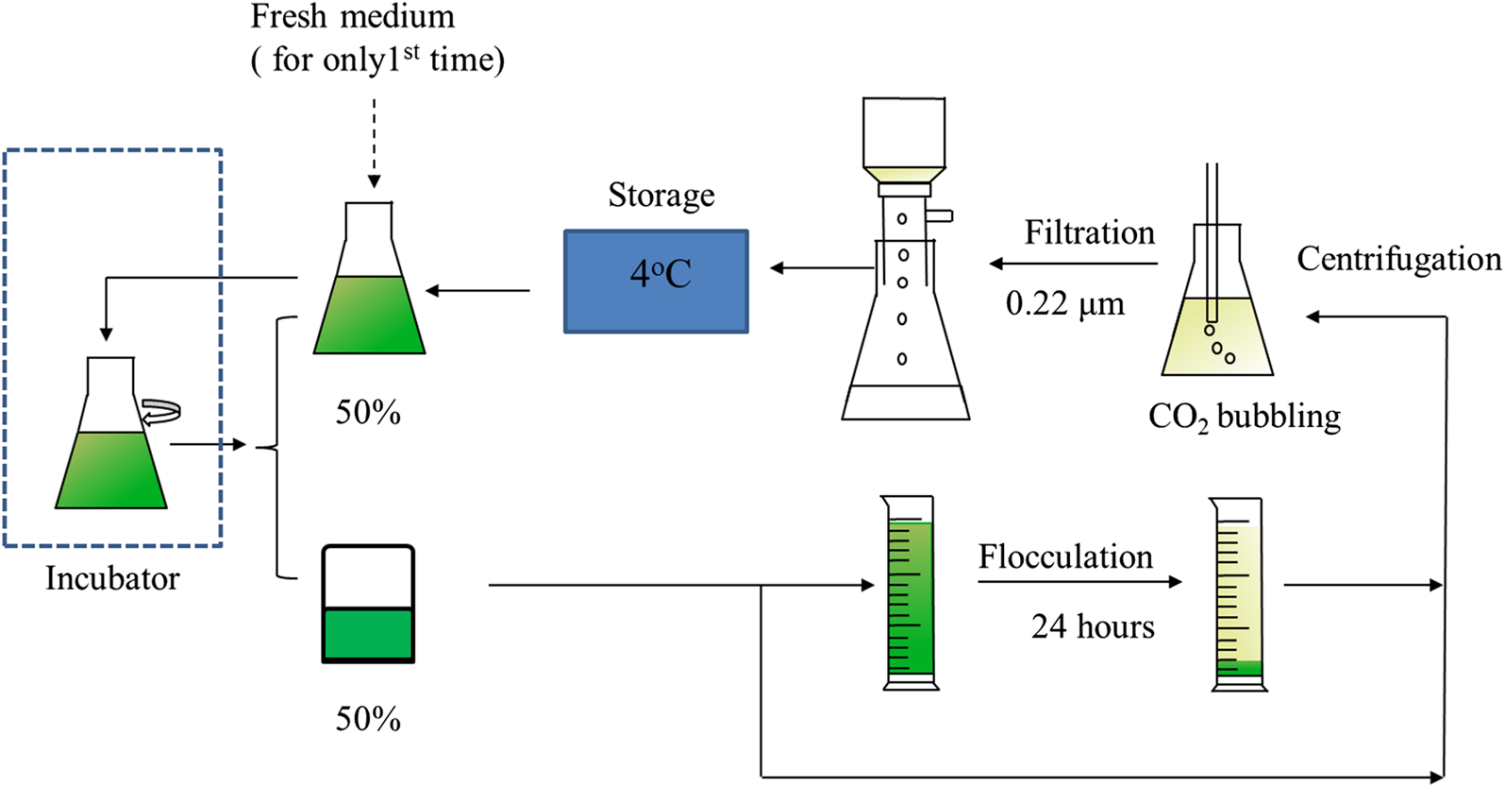
The flow diagram for a semi-continuous recycling culture of *N. oleoabundans*

To test the effect of bicarbonate concentration on growth of the adapted *N. oleoabundans* strain, batch cultures for 5 days were conducted with bicarbonate at concentrations of 0.1, 0.3, 0.5, and 0.7 mol L^−1^. Another culture was set up with a zero bicarbonate concentration that contained 0.3 mol L^−1^ sodium chloride. Both of these were conducted in a 1-L Erlenmeyer flask with a work volume of 0.4 L. To test the recyclability of spent medium, cultures grown in 3.0-L Erlenmeyer flasks were conducted in semi-continuous mode (Fig. 2) with an initial culture volume of 0.8 L. After 2 days, 50% of the culture was removed, and the same volume of fresh medium was added to re-start the culture. The removed cell suspension was then centrifuged, followed by bubbling with pure CO_2_ for pH adjustment and carbon replenishment, which usually takes approximately 20 min. When the pH of this medium decreased to 8.50, which is the same pH as fresh medium, the bubbling was stopped, and the supernatant was filtered through 0.22-μm hydrophilic membranes, and stored at 4 °C in the refrigerator. After 48 h, when the culture was finished, this CO_2_-replenished medium was used to replace 50% of the culture in the flask. The removed culture was then treated as such again. To avoid nutrient depletion, the same amounts of nutrients as in fresh medium were replenished in the recycled medium on the 8th and 14th day. To make a comparison, a group that always used fresh medium to replace 50% of the culture in the flask every 2 days was used. The cultures were continuously illuminated at 141.5 μmol m^−2^ s^−1^. The flasks were orbital shaken at 140 rpm at a temperature of 25 °C with no air bubbling. Biomass productivity (*P*_Biomass_) and specific growth rate (*μ*) were calculated as

1 $${P_{\text{Biomass}}}({\text{g}}\;{{\text{L}}^{ - 1}}\;{\text{day}}^{ - 1}) = \frac{{{\text{DC}}{{\text{W}}_2} - {\text{DC}}{{\text{W}}_1}}}{{{t_2} - {t_1}}},$$

2 $$\mu ({\text{day}}^{ - 1} ) = \frac{{\ln ({\text{DCW}}_{2} /{\text{DCW}}_{1} )}}{{t_{2} - t_{1} }},$$

where *P*_Biomass_ is biomass productivity (g L^−1^ day^−1^), *μ* is the specific growth rate (day^−1^), and DCW_2_ and DCW_1_ are the dry cell weight (DCW) at time *t*_2_ and *t*_1_, respectively.

DCW was determined according to Zhu et al. [21]. Briefly, triplicates of 40 mL samples were acidified with hydrochloric acid and then centrifuged at 10,000 rpm for 5 min at 25 °C. The algal pellets were then washed twice with a 40 mL ammonium bicarbonate solution. Finally, the pellets were re-suspended in 2 mL of ammonium bicarbonate, and this suspension was dried overnight at 105 °C until a constant weight. DCW was calculated by subtracting the empty dish weight from total weight.

### Calculation of apparent carbon utilization efficiency (%)

As the consumed carbon could be from both of initial medium or air, the carbon utilization efficiency in this study is defined as “apparent carbon utilization efficiency”, and it was calculated as follows:

3 $${\text{Efficiency}}(\% ) = \frac{{\Delta {\text{DCW}} \times C_{\text{C}} }}{{\Delta {\text{Tic}}}} \times \% ,$$

where the ∆Tic is the variation of Tic during the cultivation process and ∆DCW represents the variation in biomass concentration. *C*_C_ represents the carbon content in the dry cell weight of the algal biomass, which was measured with a combustion CHNS-analyzer (Element, Germany). In this study, the *C*_C_ of 45.7 ± 0.18, 51.6 ± 0.17, 54.7 ± 0.14 and 53.1 ± 0.01% was achieved in algal biomasses cultured with bicarbonate concentrations of 0.1, 0.3, 0.5, and 0.7 mol L^−1^, respectively. To simplify the calculation process, it was assumed that the *C*_C_ did not change during the whole cultivation process or in different culture modes when using a constant bicarbonate concentration.

Since the alkalinity of the culture media does not change with CO_2_ consumption or supply [22], total inorganic carbon (Tic) was calculated with the measured pH according to the following equation [23].

4 $${\text{Tic}} = \frac{\text{Alc}}{{\alpha_{1} + 2\alpha_{2} }},$$

where Alc is the medium alkalinity and *α*_1_ and *α*_2_ are the ionization fractions of HCO_3_^−1^ and CO_3_^2−^, respectively, which are obtained as a function of pH and the equilibrium constants *k*_1_ and *k*_2_ as shown in the following equations.

5 $$\alpha_{1} = \frac{1}{{\left( {1 + \frac{{H^{ + } }}{{k_{1} }} + \frac{{k_{2} }}{{H^{ + } }}} \right)}},$$

6 $$\alpha_{2} = \frac{1}{{\left( {1 + \frac{{H^{ + } }}{{k_{2} }} + \frac{{\left( {H^{ + } } \right)^{2} }}{{k_{1} k_{2} }}} \right)}}.$$

The equilibrium constants *k*_1_ and *k*_2_ were theoretically calculated from salinity and temperature according to Millero et al. [24].

In addition, the concentration of dissolved CO_2_ (CO_2D_) was calculated from the measured pH value and Tic as follows:

7 $${\text{CO}}_{{2{\text{D}}}} = {\text{Tic}} \times \frac{1}{{\left( {1 + \frac{{k_{1} }}{{H^{ + } }} + \frac{{k_{1} k_{2} }}{{\left( {H^{ + } } \right)^{2} }}} \right)}}.$$

In this study, the CO_2D_* is the liquid-phase CO_2D_ concentration, which is in equilibrium with the air CO_2_, and the corresponding culture pH was defined as pH*. This equation was solved using Matlab 14.0.

The CO_2_* can be calculated as follows:

8 $${\text{CO}}_{{_{2} }}^{*} = H_{{{\text{CO}}_{{_{2} }} }} P_{{{\text{CO}}_{{_{2} }} }} ,$$

where *P*_CO2_ is the partial pressure of saturated CO_2_ in the atmosphere and *H*_CO2_ is the Henry’s constant for CO_2_.

### Alkaline flocculation and measurements of recovery efficiency (RE)

Algal sedimentation was first investigated with fresh medium re-suspended algal cells, as this can well control culture conditions, such as pH and biomass concentration. To test this, algal cells were harvested by centrifugation and then re-suspended in fresh medium. This pretreatment has no significant influence on flocculation [25, 26]. To study the influence of calcium concentration on flocculation, the re-suspended algal suspension was added with different concentrations of calcium and stirred intensively at 1000 rpm for 10 min, followed by a gentle mixing of 250 rpm for another 20 min. The prepared algal suspension was subsequently allowed to settle for 60 min. In addition, the effect of biomass concentration on auto-flocculation was tested, where the re-suspended cell suspension had no pretreatment and was allowed to settle from 0.5 to 24 h. These experiments were carried out in triplicates in 50-mL graduated cylinders with a work volume of 40 mL. The biomass concentration and pH were 0.5 g L^−1^ and 10.0, respectively.

To test the repeatability of auto-flocculation in spent medium, the recovery efficiency of culture in spent medium was tested with the medium–microalgal cell mixture drawn from bath and semi-continuous mode. Neither of these cultures had any treatment. For batch culture, the cell suspension was drawn after 5 days of batch cultivation, while the recycling semi-continuous culture was drawn per 2 days. To avoid the culture loss of recycling culture, the spent medium in the semi-continuous culture was collected after the flocculation test. However, in our practical experiment, 2 mL of medium was used for sampling, and the rest was recovered by centrifugation, to avoid volume loss. The recovery efficiency was measured by calculating the absorbance difference between the initial (*C*_0_) and the final (*C*_t_) optical density at 750 nm.

The recovery efficiency (RE) was calculated as:

9 $${\text{Recovery}}\;{\text{efficiency}}(\% ) = \frac{{C_{0} - C_{t} }}{{ \, C_{0} }} \times 100,$$

where *C*_0_ is the initial optical density and *C*_t_ is the final optical density after settlement.

The concentrating factor (CF) was determined as

10 $${\text{CF}} = \frac{{H_{1} }}{{H_{2} }},$$

where *H*_1_ is the height of algal suspension in the cylinder at the beginning and *H*_2_ is the height of algal sludge at the end of sedimentation.

### Statistical analysis

Differences between groups were performed by one-way analysis of variance (ANOVA) tests. The significant differences were considered at *p* < 0.05, as determined by SigmaStat (version 3.1) software (SPSS).

## Results

### Effect of bicarbonate concentration on the growth of *N. oleoabundans*

**Fig. 3:**
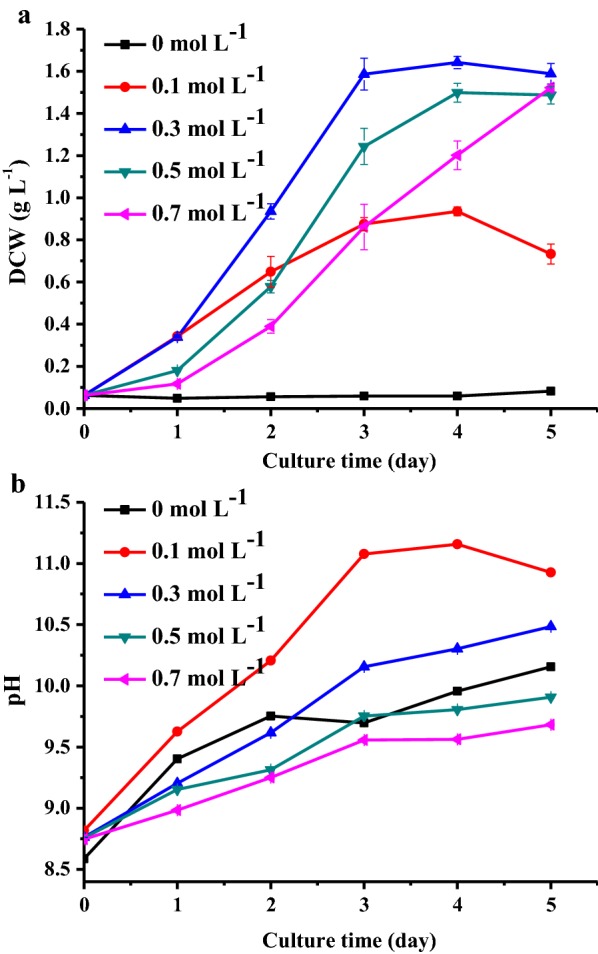
**a** Growth of *N. oleoabundans* at different concentrations of NaHCO_3_; **b** pH change during the cultivation process. All data represent the mean ± SD from three independent experiments

Biomass concentration and pH were measured during 5 days of cultivation with different concentrations of sodium bicarbonate. As shown in Fig. 3a, the algal growth with zero bicarbonate was low after 5 days of cultivation, where its final biomass density was only 0.083 g L^−1^. This is because the algal growth was limited due to low carbon concentrations. Compared with this, the bicarbonate can provide sufficient inorganic carbon for algal growth, and high biomass concentrations of 0.94 ± 0.02, 1.64 ± 0.03, 1.50 ± 0.05 and 1.52 ± 0.02 g L^−1^ were achieved in cultures with 0.1, 0.3, 0.5, and 0.7 mol L^−1^ of bicarbonate, respectively. The corresponding average volumes of productivity during the first 4 days were 0.21 ± 0.01, 0.40 ± 0.01, 0.36 ± 0.01 and 0.29 ± 0.01 g L^−1^ day^−1^, respectively, while the specific growth rates were 1.71 ± 0.03, 1.70 ± 0.03, 1.17 ± 0.04, and 1.21 ± 0.07 day^−1^, respectively. This indicates that *N. oleoabundans* can survive in bicarbonate concentrations up to 0.7 mol L^−1^, although it had optimal growth at 0.3 mol L^−1^.

Since algal growth is usually affected by pH, changes in pH during the culture process were measured. As shown in Fig. 3a, biomass concentration in the culture with 0.1 mol L^−1^ bicarbonate stopped increasing after 3 days and decreased at day 5. This should be caused by high pH limiting, as the pH of the 0.1 mol L^−1^ culture was higher than 11.2 at day 3 (Fig. 3b). For the other three groups, pH values were always less than 10.5, indicating that higher concentrations of bicarbonate/carbonate had better pH buffer effects [27].

### Effect of bicarbonate concentration on carbon utilization efficiency

**Fig. 4:**
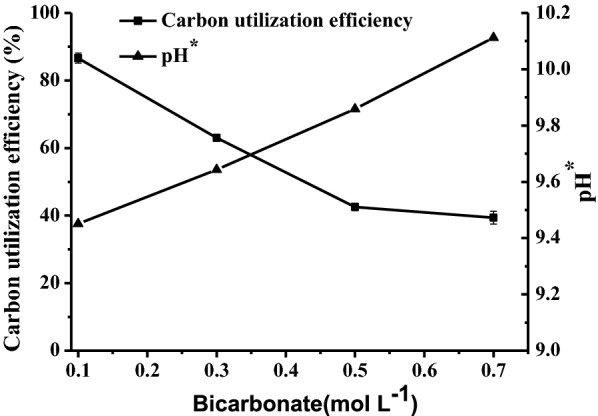
The effect of bicarbonate concentration on carbon utilization efficiency and pH*

Bicarbonate concentration significantly affected carbon utilization efficiency. As shown in Fig. 4, the apparent carbon utilization efficiency decreased with increases in bicarbonate concentration, and the overall carbon utilization efficiencies of 86.6 ± 1.5, 63 ± 1.0, 42.5 ± 1.1 and 39.4 ± 1.9% were achieved in cultures with bicarbonate concentrations of 0.1, 0.3, 0.5, and 0.7 mol L^−1^, respectively. pH* is the culture pH when the dissolved CO_2_ in the culture is equal to the saturated concentration of CO_2_ in the medium. This can be calculated through Eq. 7, and it was calculated as 9.45, 9.64, 9.86 and 10.11 for bicarbonate concentrations of 0.1, 0.3, 0.5, and 0.7 mol L^−1^, respectively. CO_2_ is transferred to the medium from the air when its pH exceeds pH*. This is the reason why higher apparent carbon utilization efficiency was achieved in the culture with 0.1 mol L^−1^, since its pH exceeded pH* after the 1st day (Fig. 3b).

### Alkaline flocculation with or without calcium

**Fig. 5:**
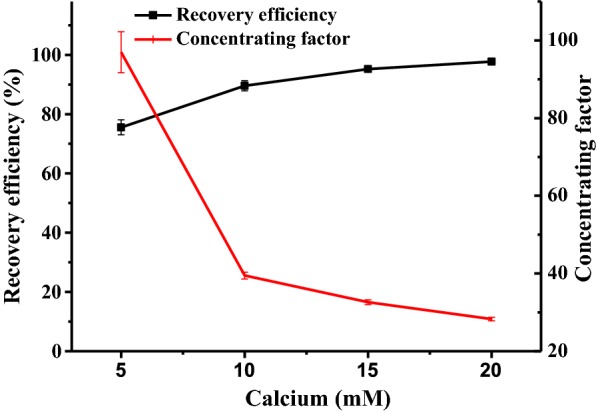
The flocculation efficiency of *N. oleoabundans* and concentrating factor at different calcium concentrations (*N. oleoabundans* biomass concentration: 0.5 g L^−1^; pH: 10.0, settling time: 1 h). All data represent the mean ± SD from three independent experiments

Figure 5 shows the harvesting efficiency with flocculation by supplement of different concentrations of calcium. On the whole, higher concentrations of calcium resulted in higher recovery efficiency. The highest was 97.7 ± 0.29%, achieved at a calcium concentration of 20 mmol L^−1^. Different from this, the concentrating factor decreased with increased dosages of calcium. The highest was 97.0 ± 5.25, achieved at a calcium concentration of 5 mmol L^−1^. A high concentration of calcium produced loose calcium carbonate precipitation, which increased the height of the algal sludge. This should be the reason why a higher calcium concentration resulted in a lower concentrating factor. A similar phenomenon was observed in another study on algal flocculation induced by magnesium [26].

**Fig. 6:**
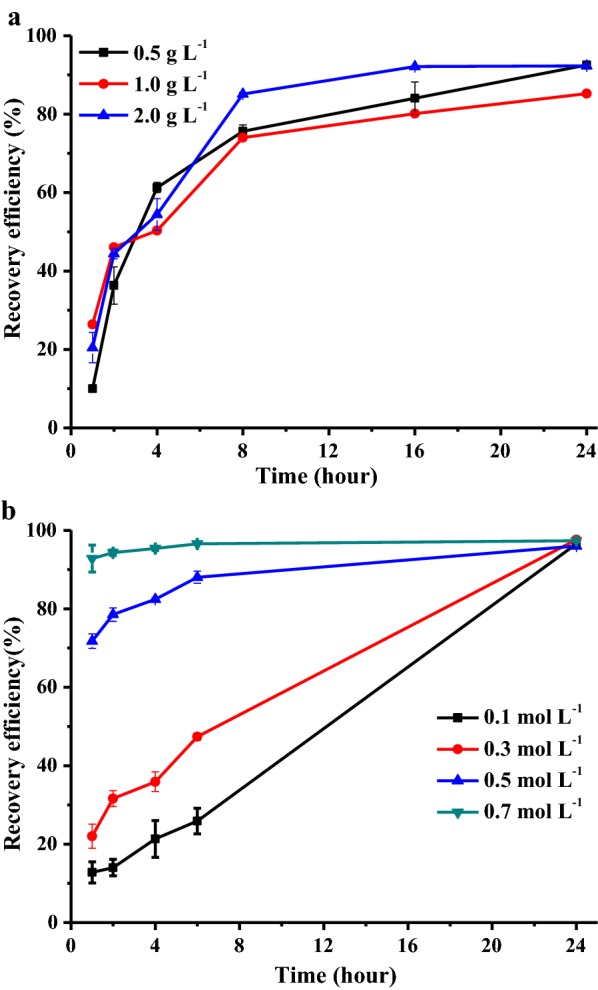
The flocculation efficiency of *N. oleoabundans* without added Ca^2+^. **a** Efficiency of auto-flocculation versus time with different biomass concentration (bicarbonate concentration: 0.3 mol L^−1^, and pH:10.0), **b** flocculation in spent medium from 5 days batch cultivation with different bicarbonate concentration. All data represent the mean ± SD from three independent experiments

For auto-flocculation without any calcium supplement, the recovery efficiency with initial biomass concentrations of 0.5, 1.0 and 2.0 g L^−1^ was investigated. As shown in Fig. 6a, recovery efficiency was less than 50% for all three concentrations in the first two hours. After 24 h, the recovery efficiency was 92.5 ± 0.62, 85.3 ± 0.01 and 92.3 ± 0.17% for biomass concentrations of 0.5, 1.0 and 2.0 g L^−1^, respectively. This indicates that auto-flocculation is an effective harvesting method for *N. oleoabundans*. However, there was no significant difference between recovery efficiencies with these three investigated biomass concentrations.

### Effect of bicarbonate concentration to auto-flocculation efficiency

In addition to affecting algal growth, bicarbonate concentration also influences the efficiency of auto-flocculation. As shown in Fig. 6b, recovery efficiency was improved with increased bicarbonate concentration in the first hour, and the highest was 92.8 ± 3.4%, achieved at 0.7 mol L^−1^. However, there was no difference in recovery efficiency for these four cultures in 24 h, where the recovery efficiencies were 96.6 ± 0.67, 97.6 ± 0.39, 96.0 ± 0.31 and 97.4 ± 0.21% for 0.1, 0.3, 0.5, and 0.7 mol L^−1^, respectively.

### Recycling culture

**Fig. 7:**
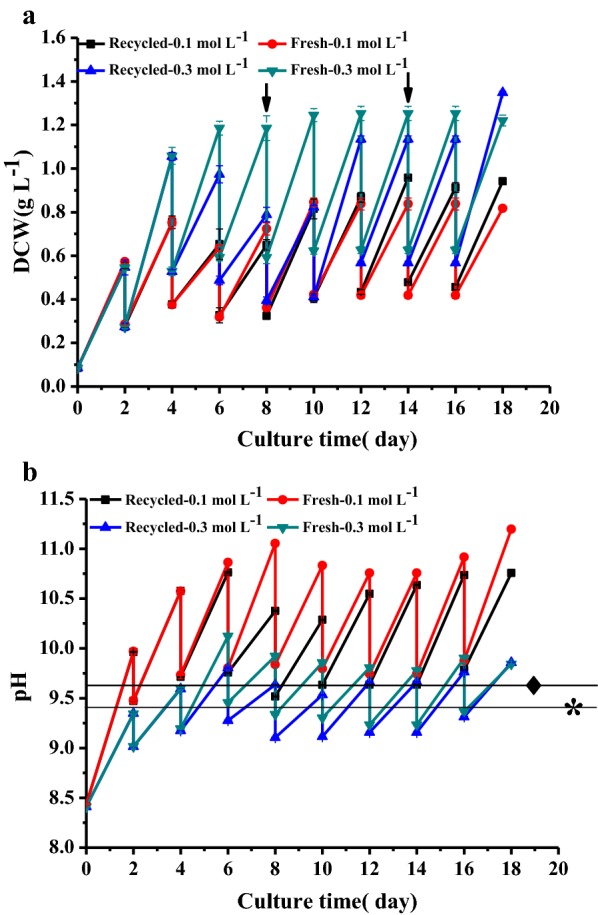
A recycling culture of *Neochloris oleoabundans* with semi-continuous mode. **a** Biomass concentration, **b** pH change (spent medium bubbled CO_2_ to adjust pH and supply carbon). Arrows mean that the nutrients were added to the recycling culture at the 8th and 14th day, while the lines with asterisk and rhombus represents the pH* of 0.1 and 0.3 mol L^−1^ bicarbonate, respectively. Values are the mean ± SD of three independent measurements

**Fig. 8:**
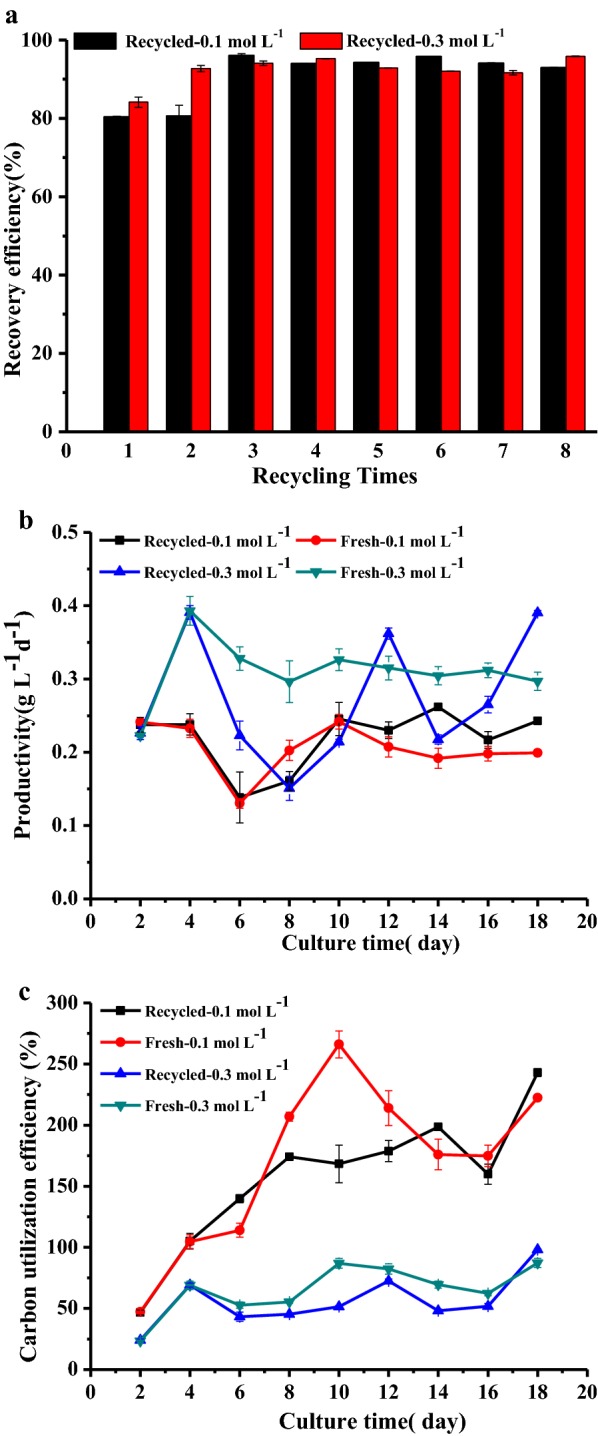
**a** Auto-flocculation harvesting efficiency of *N. oleoabundans*; **b** daily biomass productivity and **c** carbon utilization efficiency during semi-continuous culture. Values are the mean ± SD of three independent measurements

As shown in Fig. 7a, there was no significant difference in biomass concentration between fresh and recycled medium during the first 4 days for 0.3 mol L^−1^ bicarbonate and the first 6 days for 0.1 mol L^−1^. However, biomass concentration in the recycling culture with 0.3 mol L^−1^ bicarbonate was lower than its control from the 4th to 16th day. This also occurred from the 6th to 8th day for the culture with 0.1 mol L^−1^ bicarbonate, which should be attributed to nutrient depletion, since nutrient supplements added at the 8th and 14th day in the recycled medium increased the biomass productivity of the 0.3 mol L^−1^ recycling culture (Fig. 8b), while that of 0.1 mol L^−1^ recycling culture was at the 8th and 18th day. However, a small decrease in biomass productivity was found in 0.1 mol L^−1^ recycling culture at 14th day, and this reason was not clear, but both of two recycling cultures produced even higher biomass concentration than control at 18th day (Fig. 7a). This proved the feasibility of BICCAPS recycling culture.

The pH change during the entire recycling culture was recorded. As shown in Fig. 7b, the pH at harvesting time was from 9.96 to 10.76 in recycling culture with 0.1 mol L^−1^, and that of fresh medium was from 9.97 to 11.20. Compared with this, a stable pH range was found in cultures with 0.3 mol L^−1^ bicarbonate due to a stronger buffering effect. Its pH in the recycling culture was from 9.35 to 9.86, and that of the control was from 9.34 to 10.12. This showed that the pH was maintained in a stable range by replacing culture medium with recycled medium after CO_2_ absorption.

The recovery efficiency of 0.1 mol L^−1^ recycling culture was from 80.4 ± 0.1 to 96.1 ± 0.4%, and that of 0.3 mol L^−1^ recycling culture was 84.1 ± 1.3 to 95.8 ± 0.1%, as shown in Fig. 8a, indicating most algal biomass produced in this semi-continuous cultivation was successfully harvested. The biomass productivity of 0.1 mol L^−1^ bicarbonate was from 0.14 to 0.24 g L^−1^ day^−1^ for the recycling culture and that of the control was from 0.13 to 0.26 g L^−1^ day^−1^ (Fig. 8b). Compared with this, higher biomass productivity was found in the 0.3 mol L^−1^ culture, where biomass productivity in the recycling culture was from 0.15 to 0.39 g L^−1^ day^−1^, and that of the control culture was from 0.22 to 0.39 g L^−1^ day^−1^.

The carbon utilization efficiency was also measured. As shown in Fig. 8c, the carbon utilization efficiency was higher than 100% for both 0.1 mol L^−1^ cultures from the 1st day, and the highest carbon utilization efficiencies of 242 ± 3.1 and 266 ± 11% were achieved in the recycling and control culture, respectively. Different from this, the highest carbon utilization efficiency was 98 ± 0.78% in the recycling culture with 0.3 mol L^−1^ bicarbonate and 87 ± 3.6% in the control culture. The pH in both cultures with 0.1 mol L^−1^ bicarbonate exceeded the pH* from the 1st day (Fig. 7b), and CO_2_ was transferred to the medium from the air. This should be the reason why they resulted in very high carbon utilization efficiency.

## Discussion

In this study, *N. oleoabundans* was selected for semi-continuous recycling culture with BICCAPS, and this was the first proof of the recyclability of a BICCAPS. This allows a close-loop carbon capture and algal cultivation process in which sodium bicarbonate was used for microalgal culture and spent medium with a high pH and enriched carbonate was used to absorb CO_2_ for the recycling culture. Fifty percent of this culture was harvested every 2 days and the biomass productivity of the recycling culture was at the same level as the control culture, which is replaced with fresh medium. In addition, compared to normal BICCAPS without recycling, the BICCAPS with recycling can significantly decrease water usage and re-use the dissolved nutrients [28]. Auto-flocculation or calcium assisted flocculation was used for harvesting of the produced biomass, which can save the cost of expensive flocculants and avoid biomass contamination. Thus, the process developed in this study would simultaneously reduce the cost of carbon capture, salt nutrients, water, algal cultivation, and harvesting.

A higher concentration of bicarbonate is preferred for a BICCAPS, since it not only has a stronger pH buffering effect, as shown above, but also makes the CO_2_ absorption process more efficient and supplies more inorganic carbon at the beginning of each culture [27]. A lower sodium bicarbonate concentration of 0.15 mol L^−1^ was used in a previous study on the culture of *N. oleoabundans* [17, 29]. To obtain a strain that is tolerant to a high concentration of bicarbonate, the *N. oleoabundans* strain used in this study experienced an adapting process lasting for approximately 6 months. Before this adaption, it had optimal growth rate with 0.08 mol L^−1^ bicarbonate (data not shown). This indicates that an algal strains’ tolerance for a high concentration of bicarbonate may be improved by an adaption process. More alkalihalophilic strains may be obtained with this method and used in BICCAPS.

A culture with a higher bicarbonate concentration resulted in a lower carbon utilizing efficiency (Fig. 4), since it has higher pH*. CO_2_ is transferred from the air into the culture only when the culture pH is higher than pH*. Thus, the pH should be controlled at a higher level (> pH*) to achieve high carbon utilizing efficiency. In this study, the highest carbon utilization efficiency was up to 266 ± 11% in the control culture with 0.1 mol L^−1^ bicarbonate at semi-continuous mode. This is much higher than that of 91.4% in cultivation with bicarbonate at a low pH [30] and 85.6% in cultivation with 15% CO_2_ [31]. However, the high pH has a negative effect on microalgal growth. Thus, it is necessary to balance between the carbon utilization rate and the high pH’s detrimental effect. Microalgal strains tolerant to high pH are more favorable for a BICCAPS [27]. In this system, the mass transfer coefficient of CO_2_ (k_L_a_CO2_) would affect carbon utilization efficiency [32] and this should be studied in the future.

In this study, the highest growth rate was achieved in a culture with a 0.3 mol L^−1^ bicarbonate concentration, but it had a carbon cost of three times that of 0.1 mol L^−1^ bicarbonate. However, it should be noted that this high concentration of bicarbonate was used in a recycling culture rather than used in only one batch, which means that the consumed carbon will be replenished by CO_2_ gas instead of bicarbonate. Thus, the real carbon cost was the used CO_2_ gas and it was affected by the carbon utilization efficiency. Additionally, the carbon utilization efficiency of the 0.3 mol L^−1^ culture was lower than the 0.1 mol L^−1^ culture (Fig. 8c), but a high carbon utilization efficiency for 0.3 mol L^−1^ can be achieved by improving the culture pH. In addition, it also produced the same high flocculation efficiency as the 0.1 mol L^−1^ cultures (Fig. 8a). According to these results, 0.3 mol L^−1^ should be optimal bicarbonate concentration for BICCAPS to produce *N. oleoabundans* biomass with low cost. This needs to be confirmed with a comprehensive life cycle and cost assessment [33], including a consideration of biomass productivity, carbon cost, flocculation efficiency and carbon utilization efficiency. This should be conducted in our future study.

One of the advantages of BICCAPS is that the high pH resulting from bicarbonate consumption may help in algal flocculation, and this feasibility was first proved in this study. High flocculation efficiency of algal biomass was achieved with a supplement of calcium (Fig. 4). In practice, calcium can be obtained from sea water, which has a much lower cost than other flocculants that are currently used [9]. However, calcium flocculation is challenged by reversible flocculation and the potential risk of a too high Ca^2+^ content in the harvested biomass [11]. Compared with this, auto-flocculation has no such problems, and it is ideal for a BICCAPS. In this study, it was found that bicarbonate concentration plays a significant role in flocculation efficiency. The highest flocculation efficiency of 92.8 ± 3.4% was achieved in a culture with 0.7 mol L^−1^ bicarbonate at pH 9.7. The lowest flocculation efficiency of 12.8 ± 2.7% was obtained in a culture with 0.1 mol L^−1^ bicarbonate at pH 10.9. This is very different from results obtained in another study on alkaline flocculation, where higher flocculation efficiency is usually achieved in higher pH. As far as this is concerned, it may be because extracellular polymeric substances were overproduced under the stressful conditions of higher bicarbonate concentration [34], which induces efficient flocculation efficiency. More details on this would be investigated in a future study.

It is notable that auto-flocculation in some cases in this study is time-consuming (24 h) (Fig. 6). To make up for this disadvantage, semi-continuous recycling culture was used. In this mode, 2/3 of the medium was in the culture system, and the other 1/3 was in the recycling process, which includes flocculation, settling, and CO_2_ bubbling. This is also feasible in a massive-scale cultivation, since spent medium after biomass harvesting should be transported to the CO_2_ emission source to absorb more carbon and then re-transported back to the algal cultivation site. This process may take 1 or more days. However, this would not lead to a significantly higher harvesting cost, as long as enough storage tank or deep pond space is available. Additionally, a shorter harvesting time may be achieved by optimizing the pH and the initial concentration of bicarbonate (Fig. 6b), as well as the concentration of Ca^2+^, Mg^2+^, and Fe^3+^ [13, 25].

Recycling spent medium plays an important role in reducing the algal production cost. This is even more important for a BICCAPS, but it may be limited by metabolic inhibitors accumulating in the spent medium [35, 36]. However, no inhibition was observed in the whole recycling culture process of this study and it produced an even higher biomass concentration when compared with the control culture (Fig. 7a). Since no inhibition of *N. oleoabundans* growth occurred after recycling the medium so many times, inhibitor accumulation may either not occur at all or in concentrations too low to lead to inhibition. A similar phenomenon was observed in other studies, where no negative impact on the biomass growth rate or on cell quality in recycling cultures of *Chlorella pyrenoidosa* was observed [37]. A recent study also observed a positive effect in recycling of *Chlorella vulgaris* [38]. These results indicate that inhibitor accumulation and its influence is dependent on algal strains. Certainly, recycling culture is also threatened by impurities in CO_2_. For example, flue gas contains a large amount of SO_x_ and NO_x_, which may be converted into sulfate and nitrate if sodium carbonate is used to absorb CO_2_ in flue gas. In this situation, their effects to algal growth would require intensive studying in the future.

## Conclusions

The feasibility of recycling culture with BICCAPS was proved, and the biomass concentration and productivity in the recycling culture are at the same level as the control culture with fresh medium. A high carbon utilization rate of up to 266 ± 11% was obtained in fresh culture with 0.1 mol L^−1^ and 98 ± 0.78% in 0.3 mol L^−1^ recycled culture. The produced algal biomass in recycling culture was harvested with alkaline flocculation without any flocculant. These results indicated that BICCAPS is not only promising to reduce the cost of carbon capture and algal cultivation but also the cost of harvesting.
